# Will Multi-Parametric Magnetic Resonance Imaging be the Future Tool to Detect Clinically Significant Prostate Cancer?

**DOI:** 10.3389/fonc.2014.00294

**Published:** 2014-11-04

**Authors:** Gianluca Giannarini, Michele Zazzara, Marta Rossanese, Vito Palumbo, Martina Pancot, Giuseppe Como, Maria Abbinante, Vincenzo Ficarra

**Affiliations:** ^1^Department of Experimental and Clinical Medical Sciences, Urology Unit, University of Udine, Udine, Italy; ^2^Department of Medical and Biological Sciences, Institute of Diagnostic Radiology, University of Udine, Udine, Italy

**Keywords:** prostate neoplasms, magnetic resonance imaging, prostate biopsy, screening, active surveillance, radical prostatectomy

## Abstract

Multi-parametric magnetic resonance imaging is an emerging imaging modality for diagnosis, staging, characterization, and treatment planning of prostate cancer. In this report, we reviewed the literature for studies assessing the accuracy of multi-parametric magnetic resonance imaging in detecting clinically significant prostate cancer, and we critically examined the future role of this imaging tool in various clinical diagnostic settings. There is accumulating evidence suggesting a high accuracy of multi-parametric magnetic resonance imaging in ruling out clinically significant disease. Although definition for clinically significant disease widely varies, the negative predictive value is very high at up to 98%. Multi-parametric magnetic resonance imaging should, thus, be further evaluated for application in different clinical scenarios in which it is desirable to reduce the proportion of unnecessary prostate biopsies and to limit the detection of indolent disease, such as opportunistic screening, persistent prostate cancer suspicion in men with previous negative prostate biopsies, and eligibility for active surveillance. Continued improvement in standardization of technical parameters, functional sequences, and image reporting systems is a pre-requisite for a rapid and successful dissemination of this imaging modality.

## Background

In one large randomized trial, prostate-cancer (PCa) screening has resulted in a reduction in risk of metastatic disease and cancer-specific mortality (1). However, a major concern of screening is overdiagnosis of cases that would not have caused clinical consequences if left untreated (2). Overdiagnosis, in turn, leads to overtreatment, with the potential for unnecessary side effects related to curative treatments. Strategies to reduce overdiagnosis are mandatory, as are strategies to differentiate indolent from aggressive tumors. Novel biomarkers, such as serum/urine markers (e.g., PSA isoform-based “Prostate Health Index” and PCA3) and advanced imaging techniques (e.g., functional magnetic resonance imaging [MRI]) hold promise for their potential to improve accuracy in detecting clinically significant PCa. However, none of them is ready to be integrated into clinical practice because large-scale validation is lacking.

Owing to its high soft tissue contrast and high resolution, MRI provides a better visualization of the prostate and its lesions compared to transrectal ultrasound (TRUS) and other imaging modalities (3). Over the past years, its use has shifted from staging purpose to that of detection of PCa thanks to refinement in image acquisition protocols and introduction of functional techniques. The availability of higher field strength magnets (3 T), increased number of phased array receiver coils, and improved pulse-sequence techniques has resulted in a greater signal-to-noise ratio and, thus, increased spatial resolution (4). Moreover, the increasing utilization of so-called “multi-parametric MRI” (MP-MRI), deriving from the combination of conventional morphological T2-weighted sequences with at least two of the newest functional techniques, i.e., diffusion-weighted imaging (DWI), dynamic contrast-enhanced (DCE) imaging, and magnetic resonance spectroscopic imaging (MRSI), has further improved the capability to locate and characterize prostate lesions (4). DWI quantifies the microscopic mobility of water molecules in the extracellular extravascular space, providing information on cell density. PCa exhibits a reduced diffusion of water compared to normal prostate tissue due to hypercellularity with a relative decrease in water content and to disruption of interstitial spaces through which water normally diffuses. DCE imaging uses a bolus of intravenous gadolinium contrast, followed by a series of rapid sequential scans at short time intervals, to generate maps of tissue perfusion. High-grade tumors typically show an early and intense contrast enhancement followed by a rapid washout. MRSI allows for the assessment of cell metabolism by displaying the relative concentrations of citrate, choline, creatine, and polyamines. Intracellular levels of choline and creatine increase, while those of citrate decrease in malignant lesions, and are associated with tumor volume and grade (4).

Due to these peculiarities, MP-MRI has been studied in various clinical settings:

- Opportunistic screening.
- Selecting men for repeat prostate biopsy.
- Informing/guiding prostate biopsy.
- Selecting men for active surveillance.
- Monitoring tumour progression during active surveillance.
- Clinical staging.
- Treatment selection.
- Surgical planning (e.g., nerve-sparing radical prostatectomy).
- Focal therapy planning.
- Radiation therapy planning.

In two recent review articles, the use of MP-MRI-derived targets to guide biopsy has been shown to significantly improve PCa detection over systematic TRUS-guided prostate biopsy (PB), with sensitivity rates up to 80% for cancers located in the peripheral zone (5, 6). In fact, accurate lesion localization with MP-MRI enables a targeted biopsy, which overcomes the limitations inherent to the conventional systematic TRUS-guided PB, such as sampling error and undersampling of poorly accessible prostate regions (e.g., anterior gland). This high diagnostic performance is particularly evident in the setting of repeat PB after previous negative systematic TRUS-guided PB, where cancer is detected in up to 63% of patients using MRI-guidance, with up to 87% of these cancers qualifying as clinically significant (6). In addition, accumulating data suggest that the newest functional sequences, particularly DWI where a quantitative image analysis is possible, have the ability to differentiate between low- and intermediate-/high-grade tumors, at least for those cancers located in the peripheral zone (7, 8).

**Figure 1:**
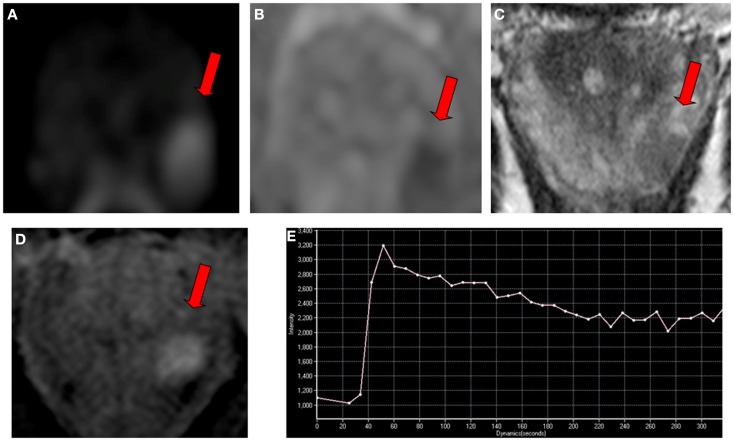
**Multi-parametric magnetic resonance imaging in a 65-year-old man referred for early detection of prostate cancer with an elevated serum PSA level (7 ng/ml) and a normal digital rectal examination**. Magnetic resonance imaging consisted of conventional T2-weighted, diffusion-weighted, and dynamic contrast-enhanced sequences performed on a 3 T unit without endorectal coil. **(A)** An ovoidal hyperintense lesion (arrow) was observed on axial diffusion-weighted imaging at *b*-value 1000 s/mm^2^ in left apical peripheral zone. **(B)** The lesion (arrow) corresponded to hypointense lesion on apparent diffusion coefficient map. **(C)** The lesion (arrow) also corresponded to axial T2-weighted image with low signal intensity, and to **(D)** focal enhanced lesion on axial dynamic contrast-enhanced imaging with a type 3 enhancement curve (washout) **(E)**. The final Prostate Imaging Reporting and Data System score for this lesion was four. The presence of a clinically significant cancer was confirmed with a targeted biopsy, showing an 8-mm Gleason score 4 + 3 adenocarcinoma.

In an attempt to standardize the reporting of MP-MRI, in 2012 an expert panel of the European Society of Urogenital Radiology (ESUR) introduced the Prostate Imaging Reporting and Data System (PI-RADS) as part of the MRI guidelines for prostate imaging (9). According to this semi-objective scoring system, each suspicious prostate lesion is assigned a point between one and five for each sequence performed as part of MP-MRI, with one being most likely clinically non-significant and five being most likely clinically significant disease. Figure 1 shows an example of clinically significant disease.

Based on all these premises, MP-MRI is a strong candidate tool for the detection of aggressive PCa, with the potential to reduce the number of unnecessary biopsies and the rate of overdetection and overtreatment.

Thus, the aim of this report is to review the literature for studies assessing the accuracy of MP-MRI in detecting clinically significant PCa, and to critically examine the future role of this imaging tool in various clinical diagnostic settings.

## Multi-Parametric MRI and Clinically Significant Prostate-Cancer: Literature Evidence

Although there is agreement that detection of “clinically significant disease” should be the primary aim of any diagnostic tool for PCa, no consensus on its definition has been reached (10). From a methodological standpoint, this is a major obstacle to assess this outcome measure in a proper and systematic manner.

Several studies are available that have been designed to address the question as to whether MP-MRI is able to in detecting “clinically significant disease.” In this non-systematic mini-review, we present only those series reporting full data on diagnostic accuracy (11–16). Details of the six included studies are reported in Table 1. Of them, four were prospective and two retrospective cohort studies. Included were patients undergoing both initial and repeat PB with varying PSA ranges. Only in one study (12), there was also a minority of men already diagnosed with low-risk PCa. Histology at PB was the reference standard in all studies. Different modalities for PB were used, ranging from transperineal template mapping to TRUS-guided sampling using cognitive or visual registration of the MRI target or registration software fusing MRI images with real-time TRUS. In addition, MP-MRI protocols and image acquisitions were not uniform, as well as statistical methods of analysis. Finally, four studies (11, 12, 14, 15) originated from the same researchers’ group, introducing a possible publication bias.

**Table 1:** **Main characteristics of the selected studies evaluating multi-parametric magnetic resonance imaging in detecting clinically significant prostate cancer and reporting full data on diagnostic accuracy**.

First author [reference]	*N*	Study design	Prostate biopsy status	Reference standard	Level of analysis for MP-MRI performance	Overall cancer detection rate (%)	Definition of clinically significant cancer	Diagnostic performance of MP-MRI for clinically significant cancer				
								Acc (%)	Sen (%)	Spe (%)	PPV (%)	NPV (%)
Rouse (11)	114	P	Biopsy-naïve	Systematic + targeted (cognitive) US-guided transrectal biopsy	Whole prostate	60	GS ≥ 7 and maximum CCL ≥ 3 mm GS ≥ 7 and maximum CCL ≥ 5 mm	64	91.5	44.8	53.8	88.2
								61.4	92.9	43.1	48.8	91.2
Arumainayagam (12)^a^	64	R	Biopsy-naïve + previous negative biopsy + previous positive biopsy	TPMB	Whole prostate/prostate halves^c^	84	UCL2 criteria	72/74/72	88/90/95	44/48/30	74/75/71	67/73/78
							UCL1 criteria	64/65/61	88/88/94	37/38/23	61/62/58	73/73/78
							Goto’s criteria	73/79/80	83/87/94	44/56/38	82/85/82	47/60/67
							Epstein’s criteria	73/79/77	85/89/94	44/56/33	80/83/78	53/67/67
							GS ≥ 7	75/73/66	100/97/100	48/47/29	67/66/60	100/93/100
Rais-Bahrami (13)^b^	583	P	Biopsy-naïve	Systematic + targeted (MRI/US fusion-guided) transrectal biopsy	Whole prostate	54	GS ≥ 7	NA	94/33	28/92	38/67	91/75
							GS ≥ 8	NA	98/45	24/89	18/41	91/91
Abd-Alazeez (14)^b^	129	R	Biopsy naïve	TPMB	Prostate halves	55	UCL2 criteria	44/68	94/68	23/69	34/48	89/83
							UCL1 criteria	42/67	98/81	22/66	21/34	98/94
							GS ≥ 4 + 3	23/62	100/92	19/61	6/11	100/99
							GS ≥ 3 + 4	36/66	93/70	21/65	24/35	92/89
							maximum CCL ≥ 6 mm	33/67	98/80	21/65	19/30	98/95
							maximum CCL ≥ 4 mm	40/68	94/81	22/67	28/42	91/88
Abd-Alazeez (15)^b^	54	P	Previous negative biopsy	TPMB	Prostate halves	63	UCL2 criteria	53/80	76/67	42/85	38/67	79/85
							UCL1 criteria	51/70	90/80	42/80	26/47	95/94
							GS ≥ 4 + 3	41/71	100/79	38/71	7/12	100/99
							GS ≥ 3 + 4	52/79	87/74	42/80	29/50	92/92
							maximum CCL ≥ 6 mm	49/78	89/77	41/78	23/41	95/94
							maximum CCL ≥ 4 mm	48/75	74/62	39/79	29/49	82/86
Thompson (16)	150	P	Biopsy-naïve	TPMB + targeted (cognitive) US-guided transperineal biopsy	Whole prostate	61	GS ≥ 7	NA	94	50	52	94
							GS ≥ 7 with > 5% Gleason grade 4		96	47	43	96
							GS ≥ 6 with ≥ 20% positive cores or maximum CCL ≥ 5 mm		93	53	58	92
							GS ≥ 6 with > 5% Gleason grade 4, ≥ 30% positive cores or maximum CCL ≥ 8 mm		96	50	50	96

*Acc, accuracy; CCL, cancer core length; Epstein’s criteria, Gleason score ≥7 or PSA density ≥0.1 ng/ml/g or ≥3 positive cores or ≥1 biopsy core with >50% involvement; Goto’s criteria, Gleason score ≥7 or maximum cancer core length ≥2 mm; GS, Gleason score; MRI, magnetic resonance imaging; MP-MRI, multi-parametric magnetic resonance imaging; NA, not available; NPV, negative predictive value; P, prospective; PPV, positive predictive value; R, retrospective; Sen, sensitivity; Spe, specificity; TPMB, transperineal prostate mapping biopsy; UCL1, University College of London definition 1 (Gleason score ≥4 + 3 and/or maximum cancer core length ≥6 mm and/or total cancer core length ≥6 mm); UCL2, University College of London definition 2 (Gleason score ≥3 + 4 and/or maximum cancer core length ≥4 mm and/or total cancer core length ≥6 mm); US, ultrasound*.

*^a^Three independent multi-parametric magnetic resonance imaging readers*.

*^b^Two different thresholds for clinically significant cancer suspicion at multi-parametric magnetic resonance imaging*.

*^c^Only data on whole prostate level analysis are reported*.

Due to all these reasons, the performance of MP-MRI in detecting “clinically significant disease” varied considerably across the studies. Accuracy, positive predictive value and negative predictive value ranged from 23 to 80%, 6 to 82%, and 47 to 100%, respectively (Table 1). It is notable, however, that the negative predictive value, albeit decreasing with higher thresholds for the definition of “clinically significant disease,” remained relatively high, implying that MP-MRI is a reliable tool to rule out potentially aggressive tumors.

## Current Challenges of Multi-Parametric MRI in Detecting Clinically Significant Prostate Cancer

The ESUR PI-RADS is based on opinion of experts, is currently undergoing changes and refinements and still awaits a formal validation (17). Such validation study should include a design where MP-MRI is performed prior to PB, several readers blinded to clinical data and with different experience in prostate MP-MRI evaluate independently the images, MRI-targeted PB is compared to systematic TRUS-guided PB, and the reference standard is final pathology of radical prostatectomy specimens with and without preoperatively known PCa so as to reduce the bias in which readers are aware that the study population comprises only patients with proven PCa. To the best of our knowledge, only a single diagnostic study on MRI used final pathology of prostates with and without cancer as the reference standard, having enrolled patients eventually treated with RP or radical cystectomy (18). This study, however, was not powered to address the issue of false positives with only 18 patients with no PCa. Moreover, only DW sequences were used. Thus, a refinement of the existing semi-objective scoring systems with focus on clinically significant disease is warranted.

Moreover, the accuracy of image reporting is strongly dependent on reader expertise. Taken the results of two recent studies in aggregate (19, 20), subjective scoring (so-called Likert scales) by experienced readers results in more accurate characterization of the likelihood of malignancy of prostate lesions seen at MP-MRI than do the semi-objective scores, such as the PI-RADS and morphology-location-signal intensity scale. It is reasonable to think that this is also the case for the characterization of clinically significant disease.

In order to disseminate in the clinical practice, MP-MRI should be standardized not only with regard to image reporting systems, but also with regard to technical equipment, examination protocols, and image acquisition, processing and post-processing. Moreover, future studies should always assess interobserver variability among expert and junior readers, and learning curve for both MP-MRI readers and operators performing MRI-informed PB (if different from readers). Communication between low-volume centers and high-volume centers should be promoted with training programs and tele-courses. Clinically significant disease should be the primary outcome measure in such studies.

Furthermore, histology parameters available at MRI-targeted biopsy may not necessarily have the same value of those available at systematic TRUS-guided PB. With the former, there is typically an upgrade in Gleason score and a higher percentage of cancer per core compared to the latter. Therefore, a new definition of “clinically significant disease” will be required. Validation of these “new” risk parameters is mandatory to determine the true value of pre-biopsy MRI.

In addition, whether MRI-targeted biopsies should be always complemented by systematic TRUS-guided PB during first and repeat PB setting remains unknown. When transperineal saturation biopsy is set as the reference standard, approximately 10% of men with “negative” MP-MRI performed by experienced high-volume radiologists still harbor intermediate-risk disease (21). It is plausible that with increasing precision in MRI-targeted biopsy technology, systematic biopsies will lose their value.

Finally, costs related to this sophisticated imaging technology are still an issue for many radiological centers worldwide. A formal cost-effectiveness analysis is eagerly awaited.

## The Future Diagnostic Role of Multi-Parametric MRI

Based on the relatively high negative predictive value for “clinically significant disease,” it might not be impossible for MP-MRI to become a first-line screening tool. This would entail a major paradigm shift in PCa. By optimizing diagnosis, and subsequently preventing overtreatment of clinically insignificant disease, MP-MRI-informed PB may provide a method for streamlining the diagnostic pathway in PCa. A recent prospective trial in PB-naïve men has provided promising results with this regard (22). Compared to systematic TRUS-guided PB, a diagnostic pathway including MP-MRI and selective MP-MRI-guided biopsy of equivocal or suspicious lesions reduced the need for biopsy by 51%, decreased the diagnosis of low-risk PCa by 89.4%, and increased the detection of intermediate/high-risk PCa by 17.7%.

With increasing adoption of scanners with higher strength fields (i.e., 3 T) ensuring a better image quality without an endorectal coil, patient acceptance is expected to increase. On the other hand, it remains to be proven whether DCE-MRI has an additional diagnostic value compared to DW-MRI with conventional T2-weighted MRI, particularly for the detection of significant disease (23). Furthermore, the use of MRSI is decreasing because it requires an endorectal coil even using high strength fields, complex software, a longer training to achieve proficient image interpretation and a long reading time. If DW-MRI alone combined with conventional T2-weighted MRI would prove to be sufficiently accurate as a biparametric technique, the advantages would be numerous. Compared to the other functional techniques, in fact, DW-MRI requires no contrast medium administration, no special software for image analysis, and no particular experience in image interpretation. Moreover, image analysis is faster and less expensive, and quantitative data can be used to predict PCa grading. Therefore, this biparametric technique could be applied to broader clinical settings, including larger patient populations, such as those for screening and early detection programs.

Active surveillance (AS) is an emerging treatment option for most low- and some intermediate-risk PCa patients with the aim of reducing overtreatment of indolent disease. Eligibility criteria in all representative AS protocols are based on standard clinico-pathological variables, which are inaccurate to predict “clinically significant disease.” The risk of misclassification is, thus, a major problem. With this regard, MP-MRI could be a useful tool both to determine initial eligibility for AS and to monitor disease progression. MP-MRI performed early after an initial standard TRUS-guided PB suggesting histological suitability for AS could exclude immediately those misclassified patients (approximately 30%) with “clinically significant disease” (24). However, it remains to be determined whether the adoption of a MP-MRI-based pathway with targeted PB is superior to, e.g., a systematic saturation PB as a reclassification tool at entry in AS protocols, the latter being clearly less expensive. Identifying tumor progression during AS is also a major challenge, given the inaccuracy of the standard clinical and histological parameters. MP-MRI could then significantly reduce the number of unnecessary surveillance biopsies, thereby making AS less invasive. Unfortunately, there is no currently accepted definition of “radiological” progression. It is likely that this will be based both on morphological parameters (e.g., lesion size/volume) and functional parameters (e.g., changes in qualitative and quantitative parameters derived from functional sequences such as DWI and DCE).

## Conclusion

MP-MRI is an emerging imaging modality for diagnosis, staging, characterization, and treatment planning of PCa. There is accumulating evidence suggesting a high accuracy of MP-MRI in ruling out “clinically significant disease.” MP-MRI should, thus, be further evaluated for application in different clinical scenarios in which it is desirable to reduce the proportion of unnecessary PBs and to limit the detection of indolent disease, such as opportunistic screening, persistent PCa suspicion in men with previous negative PB, and eligibility for AS. Continued improvement in standardization of technical parameters, functional sequences, and image reporting systems is a pre-requisite for a rapid and successful dissemination of this imaging modality.

## Conflict of Interest Statement

The authors declare that the research was conducted in the absence of any commercial or financial relationships that could be construed as a potential conflict of interest.
